# A rapid systematic review of the effect of health or peer volunteers for diabetes self-management: Synthesizing evidence to guide social prescribing

**DOI:** 10.1371/journal.pgph.0004071

**Published:** 2024-12-31

**Authors:** Thomas Iverson, Hadil Alfares, Gurkirat Singh Nijjar, Jeffrey Wong, Emaan Abbasi, Elham Esfandiari, Margaret Lin, Robert J. Petrella, Bobbi Symes, Anna Chudyk, Maureen C. Ashe

**Affiliations:** 1 Department of Family Practice, The University of British Columbia, Vancouver, Canada; 2 Department of Sociology, Trinity Western University, Langley, Canada; 3 University of Galway, Galway, Ireland; 4 Edwin S.H. Leong Centre for Healthy Aging, The University of British Columbia, Vancouver, Canada; 5 Fraser Health Authority, Surrey, Canada; 6 Center for Studies in Family Medicine, Schulich School of Medicine and Dentistry, Western University, London, Ontario, Canada; 7 United Way British Columbia, Burnaby, Canada; 8 College of Pharmacy, University of Manitoba, Winnipeg, Canada; Chulalongkorn University College of Public Health Sciences, THAILAND

## Abstract

Social prescribing is a model of care, usually in the community-setting, which aims to address people’s unmet social needs. Volunteers support primary health care and community-based care in non-medical roles. However, few studies focus on volunteers in social prescribing, therefore, aimed to synthesize the effect of health or peer volunteer-led interventions on psychosocial and behavioural outcomes for middle-aged and older adults with Type 2 Diabetes Mellitus (T2DM) to inform future work for volunteering in social prescribing. We followed Preferred Reporting Items for Systematic reviews and Meta-Analyses (PRISMA) guidelines and searched six databases and Google Scholar for peer-reviewed studies from 2013+ (last search May 16, 2024). We included randomized controlled trials (RCTs) from all languages, and synthesized data using the Cochrane’s Synthesis Without Meta-analysis (SWiM) guidelines; and assessed risk of bias using the "Risk of Bias 2 Tool". We identified nine RCTs (reported in 10 publications). Interventions aimed to promote self-management of T2DM, and study duration ranged from one to 46 months. Training for volunteers varied between one to 32 hours, and most volunteers were offered a stipend. For psychosocial outcomes, only one outcome on social support favoured the intervention group, with the remaining outcomes reporting no differences between study groups. For behaviour, six outcomes (from three studies) favoured the intervention group, and for three outcomes there were no differences between study groups. In conclusion, volunteers bring a unique perspective to health interventions, but volunteer training, matching and retention, as well as intervention mode and duration, and geographical context need to be thoughtfully considered as important implementation factors. This work generates ideas for future studies focused on volunteers and T2DM management and social prescribing.

**Trial registration: PROSPERO registration:** CRD42023453506.

## Introduction

Volunteers, often due to altruistic motives, engage in unpaid non-profit activity that contributes significantly to society [1, 2]. In a health context, volunteers most often donate their time to structured organizations such as governments and public health agencies to deliver health messaging and behaviour change interventions in community-based settings [3, 4]. They provide unique and important, but limited roles within health interventions [3, 5]. Specifically, volunteers supplement the work of integrated care teams by providing social and emotional support [3, 5], bridging the gap between health providers and communities [3], leading physical activity or educational interventions [5], and acting as the “eyes and ears” [1] (p. 60S) of a care team [1, 5]. In such positions, volunteers can be effective at initiating behaviour change [6], improving psychosocial outcomes [5, 7, 8], and potentially reducing strain on the health care system [9, 10].

There are different groups that fall within the overarching category of volunteers, and these groups often play distinct roles, filling different gaps and tackling context-specific needs [1]. Two types of volunteers that are relevant to the community health context are community health volunteers and peer volunteers. Community health volunteers (who may also be referred to as community health workers, community health champions, lay health workers, promoters or promotoras) are a type of lay health worker who lack professional health training, but have been specifically instructed to promote health within their own communities [11, 12]. They are integral, particularly in low and middle-income countries, for achieving widespread and equitable healthcare coverage [11]. On the other hand, peer volunteers are an alternative to traditional health volunteers. They have “experiential knowledge of a specific behaviour or stressor and similar characteristics as the target population” [13] (p. 329), which is presumed to lead to more effective communication, providing behavioural and psychosocial benefits to both peers [14–16] and patients [15, 17–21]. Peer volunteers can be particularly beneficial for patients with noncommunicable diseases because they are thought to enhance social support, which is linked with a patient’s ability to self-manage their disease or condition [22]. The potential for peers to enhance social support is particularly relevant as public health agencies attempt to reduce social isolation in diverse and aging populations [20]. Henceforth, we will use the term “volunteers” as an inclusive term for all health volunteers; and the term “peers” to refer specifically to peer volunteers.

Social prescribing is a community-based model of health and social care that seeks to connect individuals with non-clinical resources aimed at addressing unmet social needs, sometimes connected to living with social risk factors related to the social determinants of health [23]. It is an area supported by volunteers [24]. It originated in the United Kingdom (UK), where it is a key part of the publicly-funded drive towards Universal Personalised Care [25], but has since migrated to other countries, including Canada [23]. However, social prescribing in Canada remains a developing field that receives funding on a case-by-case basis, and programs receive noteworthy contributions from volunteers [26].

In the UK, volunteers are often relied upon to develop one-to-one relationships with service users, while paid staff oversee the programs at a higher level [27]. In addition, there are studies which focus on the role of volunteering as a social prescription [16, 28, 29]; and emerging data on volunteers or the voluntary sector in social prescribing [30–32]. However, it remains difficult to recruit and train an adequate volunteer workforce, which can be challenging for paid staff and complicates the evaluation of the roles and effectiveness of the volunteers [24, 27]. As a result, although volunteers are used regularly by social prescribing programs globally, there is limited peer-reviewed evidence documenting their roles or effectiveness within the programs [33].

Social prescribing is an all-inclusive model of care, but can be particularly effective for people with long-term health conditions and people with complex social and mental health needs [25]. For example, people with Type 2 Diabetes Mellitus (T2DM) may benefit from social prescribing, since they commonly experience psychological and social barriers that make it more difficult to manage their condition [34]; and there are specific social prescribing programs focused on people living with diabetes [35–40]. People with T2DM also benefit from diabetes self-management education and support (DSMES), which aims to “give people with diabetes the knowledge, skills, and confidence to accept responsibility for their self-management” [41] (p. 2). The driving philosophy of DSMES is notable for its focus on allowing patients to set and regulate their own goals and behaviours, rather than the goals of a health care provider [42]. In philosophy and practice, DSMES closely reflects social prescribing (e.g., one-on-one personalized care, identifying barriers, referals to non-medical services, use of volunteers, can be community-based), and importantly, there is an abundance of peer-reviewed literature discussing the roles and effect of volunteers in DSMES [41]. Due to this similarity and the abundance of evidence about volunteers in diabetes self-management, this evidence could be used to inform the implementation of volunteers in social prescribing programs.

Accordingly, the primary objective of this review was to identify evidence from randomized controlled trials (RCTs) to synthesize the effect (on patients and volunteers) of one-on-one, community-based volunteer-led interventions for middle-aged and older adults living with T2DM to help inform social prescribing research and practice.

## Methods

We conducted a rapid systematic review following standard methods, Preferred Reporting Items for Systematic reviews and Meta-Analyses (PRISMA) guidelines [43]. Our aim was to synthesize evidence for volunteers working in social prescribing. However, due to limited publications when we searched, we decided to search and summarize studies in an area which closely aligns with social prescribing: volunteers’ role in diabetes self-management education and support. We chose to conduct a rapid systematic review, which is a way to synthesize evidence using simplified or reduced methods for “the public, healthcare providers, researchers, policy makers, and funders in a systematic, resource efficient manner.” [44] (p. 3) For this rapid systematic review we followed most standard systematic review procedures, except we only included individual-RCTs, we did not conduct a forward and backward citation search of included studies, and we only included studies published within the last decade [44]. In addition, during the synthesis process, we aimed to identify information which may be relevant for end users to consider when developing volunteer training programs in social prescribing. We registered the protocol: PROSPERO registration: CRD42023453506.

### Information sources and search strategy

We searched for individual-RCTs published between 1 January 2013 and 22 November 2023 in six sources [Cochrane Central Register of Controlled Trials (CENTRAL), MEDLINE, EBSCO Databases, Embase, Epistemonikos, and Web of Science], and we also conducted an advanced search in Google Scholar (keywords in title only). We updated the search on May 16, 2024. The full search strategy is outlined in **S1 Table.**

### Selection process

Two reviewers (TI, MCA) independently screened the titles and abstracts (Level 1) to identify relevant citations in Covidence systematic review software, Veritas Health Innovation, Melbourne, Australia (available at www.covidence.org.). Next, the same two reviewers independently screened the full texts (Level 2) to identify eligible studies. The two reviewers resolved differences through discussion.

### Eligibility criteria

We used the following inclusion and exclusion criteria to define our search strategy.

#### Population

We included RCTs focused on middle-aged or older adults (mean age > 45) with T2DM. We excluded studies that included participants with pre-diabetes.

#### Intervention

We included studies providing one-on-one interventions delivered by volunteers; and included studies that did not provide compensation for volunteers, or gave volunteers a stipend or honorarium; but excluded studies where the intervention was delivered by paid health providers. We also excluded studies that described group-based interventions, or where interventions were delivered in hospitals.

#### Comparator

Any or none.

#### Outcomes

As we were interested in informing the health and social field of social prescribing (and not a specific health field, e.g., T2DM), we only included any studies that measured psychological (e.g., distress), social (e.g., social support), behavioural (e.g., physical activity), or quality of life outcomes. We also included studies that measured perceptions of the interventions, either from people receiving care or volunteers.

#### Time and type

We included individual-RCTs published in the last ten years (2013–2024).

### Data collection process

Three reviewers (TI, JW, MCA) reviewed the final list of included studies and created the data extraction form in Covidence and Excel (Microsoft Corporation, Redmond, WA, USA). One author (TI) extracted the following data, and a second author checked it for accuracy (JW): general information (author last name, year of publication, location), description of participants receiving interventions (inclusion/exclusion criteria, demographics, sample size), volunteer description (demographics, role, training, content/setting of intervention, stipend), measured outcomes, findings, and adverse events. Prior to submitting the manuscript for peer-review, two reviewers rechecked tables for accuracy (HA, GSN).

### Outcomes: Primary and secondary

Our primary outcomes included any psychological, social, or behaviour outcomes, for participants (e.g., patients) receiving interventions, or volunteers. Our secondary outcome was perceptions of the interventions, either from participants **(**receiving interventions**)** or volunteers.

### Risk of bias assessment

Two reviewers (TI, MCA) independently assessed risk of bias using Version 2 of the Cochrane risk-of-bias tool for RCTs (RoB 2) [45] to determine if there were systematic errors due to the randomization process, deviations from the intervention, missing outcome data, measurement of the outcome, and selection of the reported findings. The two reviewers resolved any discrepancies through discussion.

### Synthesis methods

Following Synthesis Without Meta-analysis (SWiM) guidelines [46], we reviewed extracted data and discussed similarities and differences across studies based on type of volunteer role, training, and outcomes. We calculated mean and standard deviation for relevant sociodemographic statistics, and then reviewed findings between intervention and control groups across studies, to summarize findings for the effect of a volunteer intervention on outcomes. Two reviewers (TI, MCA) independently reviewed and then met virtually several times to synthesize findings.

### Changes to study protocol

We made a few changes to the protocol, for example, during the early phase of developing the search strategy we refined the research questions, and target databases we would search; and decided to limit the study design to only individual-RCTs.

### Team composition and conflict of interest

Our team included university students, clinicians, people working in the non-profit sector, and researchers. None of the reviewers had authorship on any of the included studies.

## Results

### Study selection

**Fig 1:**
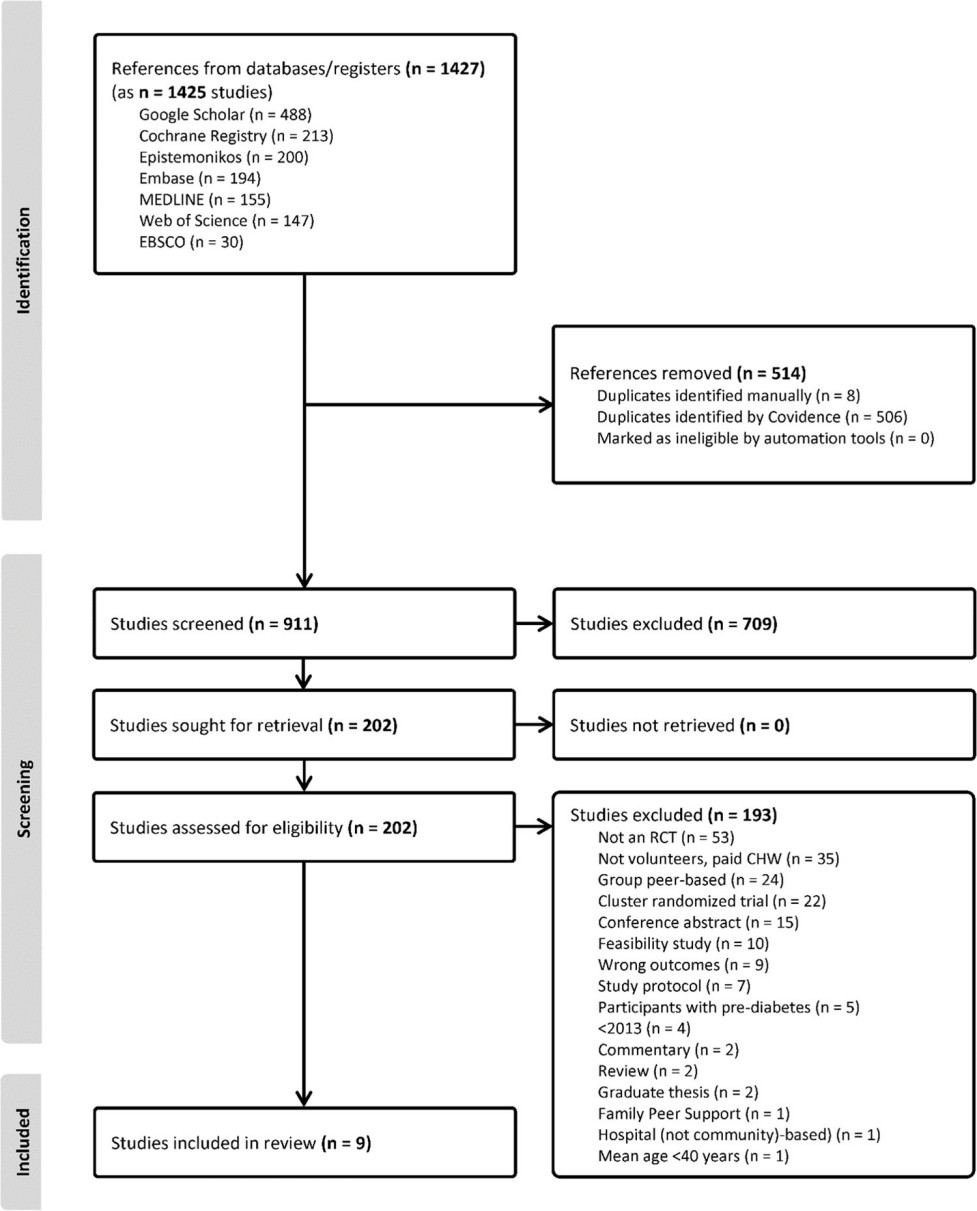
PRISMA flow diagram [43] for the review.

We identified 1427 citations (1425 studies) across sources. After removing duplicates, we screened 911 studies at Level 1 (title and abstract) and 202 studies at Level 2 (full text) review. We included nine studies (10 publications) [47–56]; and we provide a summary of the screening and selection process in **Fig 1.** The main reasons why studies were excluded were because of study design (not an individual RCT), people delivering the intervention were paid community workers, and the interventions were group-based. We excluded one study because the intervention included both group-based and one-on-one interventions [57]. We did not conduct a meta-analysis and we only reported data available within studies, or stated if data were not available. All studies were published in English.

### Study characteristics

Studies were conducted in the following locations: USA (n = 3) [47, 50, 53] and one study each from Canada [55], Hong Kong [49, 56], India [54], Malaysia [52], Taiwan [48], and United Kingdom (UK) [51]. Patients’ mean (standard deviation; SD) age was 59.4 (9.3) years, on average 38% of participants were women, and the average number of years since T2DM diagnosis was 9.1 years (6.8) (**Table 1**).

**Table 1:** Summary of patients in the intervention and control groups, and relevant outcomes.

Study Year Location	Sample, n =	N (%) Gender/Sex	Age, mean (SD)	Years with diabetes, mean (SD)	Intervention or Control Management Received	Study Outcomes
**Chan 2014 [49]** Hong Kong **Yeung 2018 [56]** Hong Kong	**Control group** (n = 316)	177 (56.0) men 139 (44.0) women	54.8 (8.6)	9.6 (7.7)	The Joint Asia Diabetes Evaluation (JADE) program (patients received personalized reports and attended a 2-hour group empowerment class)	Depression (PHQ-9) [58] Diabetes-related distress (CDDS-15) [59] Diabetes self-care activities (SDSCA1-14) [60] Diabetes self-efficacy (DES-20) [61] Medication adherence (Morisky Medication Adherence Scale) [62] Psychological distress (DASS-21) [63] Quality of life (EQ-5D) [64]
	**Intervention group** (n = 312)	178 (57.1) men 134 (42.9) women	54.5 (9.9)	9.2 (7.8)	The JADE program + the Peer Support, Empowerment, and Remote Communication Linked by Information Technology (PEARL) program (2-hour group self-care class followed by one-on-one telephonic peer support)	
**Heisler 2019 [47]** USA	**Control group** (n = 144)	142 (98.6) male 2 (1.4) female	62.1 (10.5)	15.3 (9.9)	Face-to-face and telephonic peer support. Peers had access to consumer guides containing information about diabetes self-care	Perceived diabetes-specific social support (DSS) [65]
	**Intervention group** (n = 146)	141 (96.6) male 5 (3.4) female	64.3 (9.7)	15.0 (10.2)	Face-to-face and telephonic peer support. Peers had access to iDecide, a program that generates personalized feedback while sharing the same information as the consumer guides	
**Hsu 2021 [48]** Taiwan	**Control group** (n = 33)	22 (66.7) male 11 (33.3) female	54.8 (6.9)	NA	1–2 months of non-surgical periodontal treatment (full-mouth scaling, root planning, and oral hygiene instructions provided by a dental hygienist)	Attitudes towards periodontal health [48] Oral health quality of life (OHIP-14T) [66] Oral health-related knowledge [67] Oral self-care behaviours [48]
	**Intervention group** (n = 35)	18 (51.4) male 17 (48.6) female	54.7 (6.1)	NA	Non-surgical periodontal treatment + a periodontal care curriculum taught by community health workers	
**Long 2020 [50]** USA	**Control group** (n = 154)	146 (94.8) male 8 (5.2) female	60.6 (7.4)	14.2 (8.0)	Usual care for T2DM	Depression (PHQ-2) [68] Diabetes distress (DDS2) [69]
	**Intervention group** (n = 202)	195 (96.5) male 7 (3.5) female	59.6 (7.9)	13.8 (9.1)	Usual care + telephonic peer support	
**Sampson 2021 [51]** United Kingdom	**Control group** (n = 149)	63 (42.3) male 86 (57.7) female	63.5 (10.0)	*patients were screened and newly diagnosed with T2DM	2-hour group education and behaviour change session	Diabetes quality of life (ADDQol) [70] Diabetes management self-efficacy (DMSES) [71] Diabetes treatment satisfaction (DTSQ) [72] Dietary behaviours (DBQ) [73] Physical activity (IPAQ) [74] Quality of life (EQ-5D) [75] Well-being (WBQ-12) [76]
	**Intervention group 1** (n = 142)	61 (43.0) male 81 (57.0) female	64.6 (10.1)		The Norfolk Diabetes Prevention Study (NDPS) intervention (six 2-hour group education and behaviour change sessions, followed by up to 15 2.5-hour group-based maintenance sessions)	
	**Intervention group 2** (n = 141)	57 (40.4) male 84 (59.6) female	64.1 (9.9)		The NDPS intervention + telephonic peer support	
**Sazlina 2015 [52]** Malaysia	**Control group** (n = 23)	11 (47.8) men 12 (52.2) women	63.0 (7.0)	6.0 (9.0)	Usual care based on Malaysian guidelines for T2DM management (education about lifestyle modification, medication and self-care)	Exercise self-efficacy (SEES) [77] Perceived social support (MSPSS) [78] Physical activity (pedometer, weekly duration & frequency, PASE) [79] Psychological wellbeing (GHQ-12) [80] Quality of life (SF-12) [81]
	**Intervention group 1** (n = 23)	14 (60.9) men 9 (39.1) women	63.0 (8.0)	10.0 (9.0)	Usual care + personalized feedback about physical activity patterns	
	**Intervention group 2** (n = 23)	12 (52.2) men 11 (47.8) women	64.0 (7.0)	9.0 (11.0)	Usual care + personalized feedback + face-to-face and telephonic peer support	
**Siminerio 2013 [53]** USA	**Control group** (n = 32)	15 (46.9) male 17 (53.1) female	60.0 (12.0)	NA	Diabetes self-management education (DSME) provided by certified diabetes educators	Behavioural goal tracking [53] Emotional distress (PAID) [82] Empowerment (DES-SF) [83] Satisfaction [53] Self-care behaviours (SDSCA) [60]
	**Intervention group 1** (n = 38)	16 (42.1) male 22 (57.9) female	60.0 (10.0)	NA	DSME + telephonic diabetes self-management support (DSMS) provided by certified diabetes educators	
	**Intervention group 2** (n = 36)	17 (47.2) male 19 (52.8) female	64.0 (10.0)	NA	DSME + telephonic DSMS provided by peers	
	**Intervention group 3** (n = 35)	14 (40.0) male 21 (60.0) female	60.0 (13.4)	NA	DSME + telephonic DSMS provided by practice staff	
**Sreedevi 2017 [54]** India	**Control group** (n = 26)	26 (100.0) women	51.92 (6.57)	5.1 (3.04)	Usual care for T2DM	Quality of life (WHOQOL-BREF) [84]
	**Intervention group 1** (n = 31)	31 (100.0) women	51.97 (7.40)	5.8 (2.78)	24 60-minute yoga sessions led by an instructor	
	**Intervention group 2** (n = 26)	26 (100.0) women	51.92 (8.32)	5.34 (2.75)	Weekly face-to-face and telephonic peer support	
**Tang 2022 [55]** Canada	**Control group** (n = 98)	49 (50.0) male 49 (50.0) female	58.5 (10.9)	11.0 (11.5)	Usual care for T2DM	Depression (PHQ-9) [58] Diabetes distress (DDS17) [85]
	**Intervention group** (n = 98)	49 (50.0) male 49 (50.0) female	60.5 (11.4)	12.4 (11.1)	Face-to-face and telephonic peer support	

Abbreviations: ADDQol = Audit of Diabetes Dependent Quality of Life; CDDS-15 = Chinese Diabetes Distress Scale-15; DASS-21 = Depression Anxiety Stress Scale-21; DBQ = Dietary Behaviours Questionnaire; DDS = Diabetes Distress Scale; DES-20 = Diabetes Empowerment Scale-20; DES-SF = Diabetes Empowerment Scale-Short Form; DSS = Diabetes Support Scale; EQ5D = EuroQol-5D; IPAQ = International Physical Activity Questionnaire; MSPSS = Multidimensional Scale of Perceived Social Support; OHIP-14T = Oral Health Impact Profile-14; PAID = Problem Areas in Diabetes; PASE = Physical Activity Scale for the Elderly; PHQ-2 = Patient Health Questionnaire-2; PHQ-9 = Patient Health Questionnaire-9; SDSCA = Summary of Diabetes Self-Care Activities; WBQ-12 = Well-Being Questionnaire-12; WHOQOL-BREF = World Health Organization Quality of Life Scale

Volunteers were commonly referred to as peers (meaning they also had T2DM), with the exception of a single study that described volunteers as community health workers [48]; and they filled various roles such as educators, mentors, supporters, and facilitators (**Table 2**).

**Table 2:** Summary of the volunteers and the interventions.

Study	Volunteer Description	Training	Intervention
**Chan 2014 [49]** **Yeung 2018 [56]**	Peers: people with A1c < 8.0%.	**Time commitment:** 32 hours **Content:** Tutorials, case sharing, reflections, role playing, games, and activities. Focused on mindset, empathic listening, questioning skills, and counselling skills. Addressed factors that could influence blood glucose level (e.g. diet, exercise), self-monitoring of blood glucose, sick day management, foot care, emotional support, resources for information, and clinical care. **Materials:** Tutorial notes, reference materials **Ongoing supports:** NA **Assessment:** Pre- and post-training evaluation of diabetes knowledge and psychological-behavioural measures	**Length of intervention:** 12 months **Time commitment:** 2-hour face-to-face session, 15 minutes ≥ 12 phone calls **Volunteer role:** NA **Patients per volunteer:** 10 **Volunteer-patient matching:** NA **Materials:** Resource booklet, checklist for calls **Mode:** Telephone **Stipend amount:** US $500
**Heisler 2019 [47]**	Peers: people with a history of poor glycemic control (A1c ≥ 8.0%), but whose most recent A1c was < 8.0%	**Time commitment:** 2 hours (base training), 1 hour (iDecide training), 1.5 hour/month (follow-up meetings) **Content:** Motivational Interviewing-based communication skills (open-ended questions, rolling with resistance, eliciting ’change-talk’, and goal-setting and ’action planning’) **Materials:** iDecide (personally tailored diabetes medication decision aid) on iPads **Ongoing supports:** Monthly meetings with other peers to check-in and provide booster follow-up training **Assessment:** Self-assessment and random observation of phone calls by the study team	**Length of intervention:** 6 months **Time commitment:** 2-hour face-to-face session, ≥ 1 phone call/week **Volunteer role:** During the initial face-to-face session, peers and patients identified a behavioural goal and an action plan and generated a list of questions and concerns for health care providers. During follow-up phone calls, peers and patients brainstormed solutions to barriers and set new goals and action steps **Patients per volunteer:** 1–5 **Volunteer-patient matching:** NA **Materials:** Diabetes medication guides, iDecide **Mode:** Face-to-face, telephone **Stipend amount:** NA
**Hsu 2021 [48]**	Community health workers: four people selected from the community.	**Time commitment:** 4 hours **Content:** Periodontal disease and care, teaching and communication skills **Materials:** Training manual containing goals and contents of each lesson **Ongoing supports:** CHWs could contact research staff for support in the month prior to the intervention **Assessment:** Community health worker certification test	**Length of intervention:** 1 month **Time commitment:** 30 minutes x 4 face-to-face lessons **Volunteer role:** Peers taught patients about effective toothbrushing methods and tools **Patients per volunteer:** NA **Volunteer-patient matching:** NA **Materials:** Slide presentations, toothbrushing tools **Mode:** Face-to-face **Stipend amount:** US $70 per patient
**Long 2020 [50]**	Peers: patients with a history of poor glycemic control, but whose most recent A1c was ≤ 7.5% OR previous study mentees (with no A1c restriction).	**Time commitment:** 1 hour **Content:** Mentee’s story/motivations (to help set realistic goals and provide support), dealing with failure in an accepting manner, role-playing exercises, and sample questions **Materials:** NA **Ongoing supports:** Staff contacted peers 1 time/month to check in and provide training reinforcements **Assessment:** NA	**Length of intervention:** 6 months **Time commitment:** ≥ 1 phone call/week **Volunteer role:** NA **Patients per volunteer:** 1 **Volunteer-patient matching:** Based on age, race/ethnicity, sex, and insulin use. **Materials:** NA **Mode:** Telephone **Stipend amount:** US $20 per month for each month peers attempted to contact their assigned mentee
**Sampson 2021 [51]**	Peers: people with T2DM.	**Time commitment:** 14–17.5 hours **Content:** Impact of physical activity, diet, pre-diabetes, and lifestyle on T2DM. Also role-playing exercises **Materials:** NA **Ongoing supports:** NA **Assessment:** A mock call where the senior research associate assumed the role of trial participant to test peers in specific situation	**Length of intervention:** 46 months **Time commitment:** ≥ 18 phone calls. **Volunteer role:** Peers and patients discussed progress, goal achievement, action planning, and barriers to coping **Patients per volunteer:** ≤ 7 **Volunteer-patient matching:** NA **Materials:** NA **Mode:** Telephone **Stipend amount:** £350
**Sazlina 2015 [52]**	Peers: older adults (≥ 60 years) with successfully managed T2DM who lived in the same community as participants.	**Time commitment:** 2 days **Content:** Interactive discussions, simulations, and role-plays to improve peers’ ability to provide support through telephone and face-to-face contacts **Materials:** NA **Ongoing supports:** Two fortnightly and two monthly debriefing meetings over the 12-week intervention **Assessment:** Assessments by the research team at monthly clinic visits with their peers	**Length of intervention:** 3 months **Time commitment:** 3 face-to-face meetings, 3 phone calls **Volunteer role:** Peers and patients discussed barriers and motivations, and peers encouraged patients to become empowered to increase their physical activity to self-manage their diabetes **Patients per volunteer:** 3–5 **Volunteer-patient matching:** NA **Materials:** NA **Mode:** Face-to-face, telephone **Stipend amount:** NA
**Siminerio 2013 [53]**	Peers: patients who had previously attended diabetes self-management education selected based on their communication skills and willingness to participate (no A1c restriction).	**Time commitment:** 2–3 hours **Content:** Information about active listening, empowerment, and behavioural approaches. Also role-playing exercises to practice skills **Materials:** NA **Ongoing supports:** Peers could contact a Certified Diabetes Educator or their Primary Care Provider **Assessment:** Human subject modules of the associated universities	**Length of intervention:** 6 months **Time commitment:** ≥ 5 phone calls **Volunteer role:** Peers and patients engaged in a patient-centred discussion regarding the patient’s behavioural goal and barriers to achieving their goal **Patients per volunteer:** NA **Volunteer-patient matching:** NA **Materials:** Telephone scripts, behavioural goal forms **Mode:** Telephone **Stipend amount:** NA
**Sreedevi 2017 [54]**	Peers: three people with T2DM (RPG < 250 mg/dL) selected from the community based on their adherence to treatment, and capacity to be a successful mentor.	**Time commitment:** 2 days **Content:** A physician explained diabetes, glycaemic control, and medications and their synergies with physical activity. A nutritionist explained the nutritional aspects of diabetes. A psychologist provided training in communication skills, empathy, and confidentiality **Materials:** Training manual (based on peers for progress handbook) **Ongoing supports:** NA **Assessment:** NA	**Length of intervention:** 3 months **Time commitment:** 45–60 minutes x 1 face-to-face meeting/week, 1 phone call/week **Volunteer role:** During the initial session, peers collected treatment details and went over the functions of peer support. During follow-up sessions, peers and patients discussed diet, exercise, medication, emotional stress, diabetes symptoms, foot care, and more. During the final sessions, the peer conducted a final process assessment **Patients per volunteer:** 13–14 **Volunteer-patient matching:** NA **Materials:** Diary (for patients) **Mode:** Face-to-face, telephone **Stipend amount:** NA
**Tang 2022 [55]**	Peers: patients with A1c < 8.0%.	**Time commitment:** 30 hours **Content:** Knowledge, skills, and strategies to address (1) assistance in daily self-management, (2) social and emotional support, and (3) linkage to clinical care. In particular, skills in motivating and empowering patients, active listening, goal-setting and action planning, and problem solving **Materials:** NA **Ongoing supports:** NA **Assessments:** Formative and summative assessments of five domains: diabetes-related knowledge, empowerment-based facilitation, active listening, goal-setting, and perceived self-efficacy. Also ’spot check’ phone calls made by the research team to peers	**Length of intervention:** 12 months **Time commitment:** 1 face-to-face meeting, 29 phone calls **Volunteer role:** Peers and patients discussed challenges, feelings and questions about self-management, solved problems, and set goals and develop action plans **Patients per volunteer:** NA **Volunteer-patient matching:** Based on gender and geographical proximity. **Materials:** NA **Mode:** Face-to-face, telephone **Stipend amount:** CAD $400 following training, then $20 per participant per month [86]

Abbreviations: CHW = Community Health Worker; NA = Not Available; T2DM = Type 2 Diabetes Mellitus

Study interventions were intended to promote patients’ ability for T2DM self-management in order to improve cardio-metabolic function and psychosocial well-being (**Table 1**). One study specifically aimed to improve oral health for patients with T2DM [48]. For our outcomes of interest, we extracted the following data: depression [48–50, 56], dietary behaviours [51], distress [49, 50, 53, 55, 56], empowerment [53], perceived social support [47, 52], physical activity [51, 52], quality of life [48, 49, 51, 52, 54, 56], self-care behaviours [48, 49, 53, 56], self-efficacy [49, 51, 52, 56], treatment satisfaction [51, 53], and well-being [51, 52] (**Table 1**). There were no studies which reported the effect or impact on the volunteers for the outcomes of interest for this review.

Three of the included studies discussed adverse events [50–52]; but there were no reported serious adverse events related to interventions.

### Risk of bias studies

**Fig 2:**
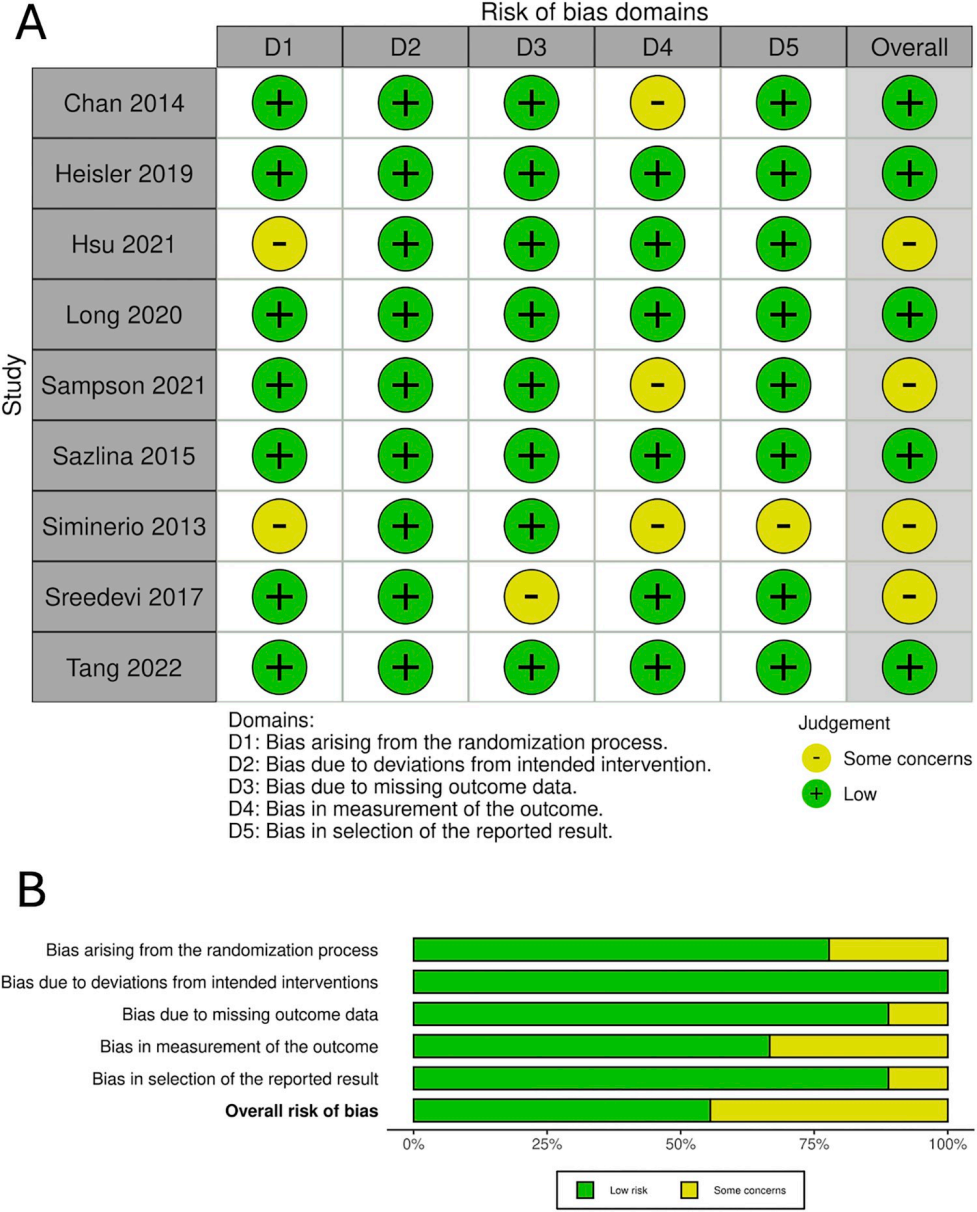
Risk of bias. (A) Summary of risk of bias for each study. (B) Overall risk of bias.

Overall, we rated five studies as low risk of bias [47, 49, 50, 52, 55], and some concerns for four studies [48, 51, 53, 54] **(Fig 2A and 2B).** Items where there were some concerns within studies included: measurement outcomes (n = 3), randomization (n = 2), missing data (n = 1), and selection of reported outcomes (n = 1).

### Results of syntheses

We provide details of the volunteer interventions in **Table 2**. The implementation of the programs varied in duration, contact time (dose), delivery mode, intervention content, volunteer training, and outcomes. The interventions ranged in duration between one and 46 months [mean (SD): 10.6 (13.8) months] and consisted of face-to-face meetings and/or telephone calls between volunteers and patients. Seven of the nine studies reported the content of their interventions, including: goal setting and action planning [47, 51, 53, 55], self-management education [48, 52, 54, 55], problem-solving [47, 51–53, 55], and discussions centred around empowerment and motivation [52]. In two of the nine studies [50, 55], researchers used pre-defined criteria to match volunteers with patients. In the remaining studies, the authors did not report how volunteers were matched with patients. Five of the nine studies [47, 49, 51, 52, 54] reported the number of patients matched with each volunteer: this number ranged from 1:1 to 1:14.

All included studies provided mandatory training for volunteers (**Table 2**.) The time commitment varied depending on the study, between one and 32 hours of training [mean (SD): 12.5 (11.9) hours], assuming that one full day of training is equal to eight hours. The training programs aimed to improve volunteers’ skills and knowledge in several areas: communication and listening [47–49, 53–55], disease self-management [48, 49, 51, 54, 55], behaviour change and goal-setting [47, 50, 51], and emotional and social support [50, 52, 53, 55]. Several studies employed role-playing in order to reinforce taught concepts [49–53].

Eight studies reported providing volunteers with a stipend to cover costs associated with participation in the study as well as to provide compensation for their substantial commitments. Of the eight studies, five specified the amount or rate of the stipend: US $500 [49]; US $70 per patient [48]; US $20 per month volunteers attempted to contact their mentee [50]; £350 [51]; and CAD $400 following training, then $20 per participant per month [55, 86]. Three studies reported providing a stipend, but not the amount [47, 52, 53]. Only one study did not report providing a stipend [54]. These results are summarized in **Table 2**.

**Results from main analyses. Table 3**provides a summary of findings for our outcomes of interest. Of 15 reported psychosocial outcomes, only one outcome (from one study) favoured the intervention group, and in 14 outcomes, there was no difference between the volunteer intervention and control groups. Of nine reported behavioural outcomes, six favoured the intervention group, and three studies reported no difference between groups.

**Table 3:** Overview of findings for outcomes of interest, comparing intervention groups who engaged with volunteers with a control condition.

Outcome Type	Favors Volunteer Intervention Group	No Difference between Groups
**Psychosocial**	Perceived social support (MSPSS) [52]	Depression (PHQ-2) [50] Depression (PHQ-9) [49, 55] Diabetes distress (DDS) [50, 55] Diabetes quality of life (ADDQol) [51] Diabetes self-efficacy (DES-20) [49] Diabetes-related distress (CDDS-15) [49] Emotional distress (PAID) [53] Empowerment (DES-SF) [53] Oral-health quality of life (OHIP-14T) [48] Perceived diabetes-specific social support (DSS) [47] Psychological distress (DASS-21) [49] Psychological wellbeing (GHQ-12) [52] Quality of life (EQ-5D) [49, 51] Quality of life (WHOQOL-BREF) [54] Well-being (WBQ-12) [51]
**Behavioural**	Attitudes towards periodontal health [48] DBQ fat score scale at 24 months [51] Oral health-related knowledge [48] Oral self-care behaviours [48] Physical activity weekly duration & frequency (PASE) [52] Steps (pedometer) [52]	Medication adherence [49] Physical activity (IPAQ, resistance questionnaire) [51] Self-care behaviours (SDSCA) [49, 53]

Abbreviations: ADDQol = Audit of Diabetes Dependent Quality of Life; CDDS-15 = Chinese Diabetes Distress Scale-15; DASS-21 = Depression Anxiety Stress Scale-21; DBQ = Dietary Behaviours Questionnaire; DDS = Diabetes Distress Scale; DES-20 = Diabetes Empowerment Scale-20; DES-SF = Diabetes Empowerment Scale-Short Form; DSS = Diabetes Support Scale; EQ5D = EuroQol-5D; GHQ-12 = General Health Questionnaire-12; IPAQ = International Physical Activity Questionnaire; MSPSS = Multidimensional Scale of Perceived Social Support; OHIP-14T = Oral Health Impact Profile-14; PAID = Problem Areas in Diabetes; PASE = Physical Activity Scale for the Elderly; PHQ-2 = Patient Health Questionnaire-2; PHQ-9 = Patient Health Questionnaire-9; SDSCA = Summary of Diabetes Self-Care Activities; WBQ-12 = Well-Being Questionnaire-12; WHOQOL-BREF = World Health Organization Quality of Life Scale

#### Results from additional analyses

Chan and colleagues [49] reported peer support management (education and peer support) had no significant effect when compared with the control group management (education alone). However, in a sub-group analysis, which controlled for patients with elevated distress at baseline [Depression, Anxiety and Stress Scale—21 Items (DASS-21) ≥ 17], peer support improved distress and medication adherence, and reduced hospitalization when compared with the control group [49]. Second, Heisler and colleagues [47] compared two peer support groups. While there was no between-group difference, both groups had improvement in diabetes-specific social support [47]. Third, Siminerio and colleagues [53] reported a comparison between three groups who received diabetes self-management support from a person from one of the following groups: a certified diabetes educator; a peer; or a practice staff member. The authors reported the intervention delivered by certified diabetes educators resulted in significantly better empowerment scores compared with delivery by peers or practice staff [53]. Finally, Sampson and colleagues [51] measured diabetes management self-efficacy, but because their participants were newly diagnosed at the time of randomization, the authors did not include a baseline diabetes management self-efficacy measurement. In their exploratory analysis, the authors reported no significant unadjusted differences between groups at 12 and 24 months for this outcome.

Two of the nine studies measured satisfaction for patients alone [51] or volunteers and patients [53] (**Table 2**). Sampson and colleagues [51] used the Diabetes Treatment Satisfaction Questionnaire (DTSQ), but did not measure satisfaction at baseline, precluding them from adjusting their data for analysis. However, the authors reported no significant unadjusted differences between groups at 12 and 24 months. On the other hand, Siminerio and colleagues [53], who using a proprietary satisfaction survey, reported patient satisfaction of 100% in the educator group, 95% in the peer group, 75% in the practice staff group, and 74% in the usual (control) group. The authors also reported that peers felt satisfied with their training and experience, and would recommend being a peer to others.

## Discussion

In this rapid systematic review, we identified nine RCTs from middle and high-income countries examining the effect of one-on-one, community-based volunteer-led interventions on the health and wellbeing of people living with T2DM, with the goal of informing practice and future research in social prescribing. Eight of the nine studies used peer volunteers [87, 88]–who were themselves living with T2DM–to provide self-management support that is often missing for people without well-developed personal support networks. Volunteers were trained in areas such as communication, behaviour change, and social support to deliver diabetes self-management interventions. However, the implementation of the programs varied in duration, contact time (dose), delivery mode, intervention content, volunteer training, and outcomes. Based on a qualitative summary of reported outcomes for participants, interventions did not have a substantial effect on psychosocial endpoints (e.g., depression, distress, quality of life). However, there were reported findings favouring volunteer interventions for social support and behaviour change, which generate hypotheses for future discovery. Overall, this rapid systematic review sheds light into how volunteers may be integrated into, and provide a meaningful contribution to, health and social models of care.

### Social support and health

The results from this review highlight volunteers play an important role in health interventions, specifically via social support and/or other behaviour change strategies [89]. The included studies did not provide specific information on the behaviour change techniques (BCTs) used by volunteers, but there were instances of goal setting, action planning and social support. Researchers have defined several different types of social support, such as unspecified, practical, or emotional [89]. Other work has outlined five possible categories of social support in health interventions, specifically personal connection, support with activities of daily living, social and emotional support (including developing coping plans and strategies), navigation, and longitudinal support [90]. In this review, included studies did not provide detailed information on the type of social support, and only two of nine studies reported social support as an outcome. However, most likely, many volunteers provided support across most of the categories of social support, except for longitudinal support, as six of the nine studies were six months or less in duration.

Many of the studies were also not designed to compare the effect of volunteer vs. provider led programs, and therefore, we cannot comment on this directly. However, in a subgroup analysis, one study [53] compared volunteers and other study personnel (e.g., pooled data from several groups) with a health provider giving the same intervention: patient empowerment scores were significantly better from the group who received the intervention from the providers. This result may not be surprising, as volunteers may not have the same longitudinal care history with patients (as providers) so it may be challenging to establish the same rapport. In addition, there may be times when people prefer information from a provider compared with a volunteer [15]. That is, the timing and type of information delivery may be context and person dependent.

### Implementation of volunteer programs

The lack of significant improvement in psychosocial outcomes due to volunteer support may be surprising. However, there are potential implementation factors to consider in these findings and in future interventions and research, such as: volunteer training, volunteer-patient matching, volunteer retention, intervention mode and duration, and geographical context.

Many volunteers do not have experience in the health and/or social care domain, and as a result they require adequate (consistent) training to equip them with the requisite skills and knowledge to be successful in their roles. What counts as adequate training is context and person dependent. In addition, training programs should be mindful that they do not require volunteers to commit too much time [91]. There was considerable variability in the length of training provided for volunteers in the included studies (1–32 hours). Despite this variability, positive behavioural results were observed for studies across the spectrum of training duration [48, 51, 52]; but not most psychosocial outcomes. However, without standardized training protocols and performance evaluations, these findings may not be generalizable or scalable. Future studies should consider implementing (and reporting) standardized training and evaluation to improve the consistency of outcomes.

Matching volunteers and patients can be completed randomly, or based on selective criteria (e.g., shared interests, geographic proximity, age, gender) which can improve rapport, and potentially lead to better outcomes for both volunteers (e.g., retention) and patients [20]. There may also be the need to reassign volunteers and patients to improve rapport [20]. One relevant matching criterion is disease or condition, which may be why eight of the nine studies in this review used peers (who, like patients, had T2DM) [47, 49–55]. In addition, only two of the included studies [50, 55] reported using additional volunteer-patient matching criteria (beyond disease or condition). However, neither study reported operational details for matching, nor reported social support outcomes. Future studies should consider defining (and reporting) criteria for volunteer-patient matching combined with ongoing evaluation of the volunteer-patient relationship (using social support or other measures) in one-to-one volunteer support programs.

Volunteers need to be recognized and valued; which may help with the sustainability of volunteer programs. Although a 2023 meta-analysis [92] identified values as the strongest predictor to volunteer; the authors also suggested understanding motivation to volunteering and regular communication [92] may support volunteer retention. Another factor which may influence the retention of volunteers in a health program is financial compensation [93]. Although they are not typically paid employees, volunteers might receive stipends to incentivize their commitment and cover basic costs incurred by their role, such as transportation [94]. Almost all of the included studies in this review provided a monetary reward to their volunteers, which can improve volunteer retention and perceived benefits [95], and may not ‘crowd-out’ intrinsic motivation [96]. Although improving volunteer retention may lead to more sustainable programs, there is a need for further research exploring whether monetary rewards have a measurable effect on patient outcomes, especially as they may modify the balance between intrinsic and extrinsic motivators. Given the current lack of evidence, our findings may not be generalizable to volunteer programs that do not provide a monetary reward.

Another important consideration when implementing a volunteer support program is the delivery mode and duration of the intervention. Previous work has shown that, while non-face-to-face contact can be beneficial in times when face-to-face contact is not possible (e.g., during COVID-19 lockdowns) [97], face-to-face contact is more effective at promoting wellbeing [98]. However, face-to-face contact can be time-consuming for volunteers and patients, depending on the meeting location, although this barrier could be overcome by matching volunteers with patients based on geographic proximity [20]. Perhaps, this is why only one of the included studies used exclusively face-to-face contact, while three studies were telephonic, and five studies used a combination of telephone and face-to-face contact. There was also considerable variability in the duration and frequency of the interventions, with studies ranging from one to 46 months in length, and meetings occurring weekly to once every two months. With positive behavioural results being observed at both ends of this spectrum, and in studies that were exclusively face-to-face and exclusively telephonic, it remains unclear what is the optimal format for intervention delivery. As a result, this rapid systematic review generates questions about the feasibility, acceptability and effects of delivery mode and duration of volunteer support interventions.

Volunteer interventions occur within local contexts, and so geographically variegated factors such as social and cultural norms, as well as healthcare systems must be considered during implementation. The studies included in this synthesis originated from a range of middle- and high-income countries, and across three continents (Asia, Europe, North America). Comparative research has attempted to classify healthcare systems by numerous variables, including expenditure, effectiveness, institutional boundaries, and disparities, but clear consensus has not been reached [99, 100]. Similarly, cultural variables are known to impact health in various ways. For example, work in Europe has shown that loneliness may have a greater negative impact on health in less individualistic, more collectivistic societies [101]. These (and other) variables may have influenced outcomes in the included studies, and we thus emphasize caution in generalizing findings across nations. Therefore, volunteer interventions should be adapted to local cultural contexts to enhance their effectiveness and produce valuable, context-specific evidence, although this warrants further investigation.

### Volunteering and social prescribing

From the start of this review, our goal was to synthesize evidence on volunteers working in social prescribing. However, despite the important role of volunteers contributing to the success of social prescribing, few peer-reviewed studies are available. From this rapid systematic review, there are potential areas to explore related to implementation, for example, considering factors for volunteer recruitment, training, sustainability, and delivery and duration; as well as consistent evaluation of volunteer training, learning, effect on patients’ health and well-being, and impact of the program on volunteers. Importantly, “success” from the perspective of the volunteer (across outcomes) should be defined in collaboration with volunteers, and routinely evaluated and consistently reported.

### Strengths and limitations

This work has many strengths. For example, we identified knowledge gaps for volunteers in health and lifestyle management; we conducted the study in accordance with PRISMA guidelines; and we built in redundancies by ensuring two reviewers completed key tasks independently, and that a second author reviewed all extracted data for accuracy. We also acknowledge several limitations with this study. For example, as a result of our narrow inclusion criteria, only nine studies (10 publications) were included in our review despite the large number of potential studies: A majority of potential studies were excluded due to study design, interventions delivered by professionals (not volunteers), or group-based interventions. However, we narrowed our inclusion criteria for several key reasons. Notably, we only included one-on-one interventions to replicate many social prescribing settings more closely, where care is typically delivered to individuals rather than groups [102, 103].

Another limitation of our review was the considerable variety of interventions and outcomes in the included studies. In particular, the control groups in the included studies received varied management strategies. Some groups received ‘usual care’, some received educational interventions that were also delivered to the intervention group, and others received alternate interventions. As a result, it was difficult to isolate the effect of volunteers from the effects of the alternate or supplemental interventions offered to patients. For instance, in a program where volunteer support is offered in addition to an educational program, it is possible that the educational program has already maximized the potential benefits, leaving little room for volunteers to provide any additional benefit. For this reason, it is important that future studies aim to standardize control group management, although we acknowledge that there will be inevitable variability owing to differences in local health context.

Finally, studies reported a wide variety of psychosocial and behavioural outcomes, and used a variety of scales and measures to record these outcomes. This heterogeneity limited our ability to combine data quantitatively and make general conclusions about the effect of volunteer interventions. We suggest future studies aim to standardize the outcomes used to assess volunteer interventions so that results can be directly compared and broader conclusions can be drawn. We also suggest that social support should be more regularly reported, as it may provide insights into the quality of the volunteer-patient relationship.

## Conclusion

In this review, we addressed a knowledge gap and highlight the potential role of volunteers to support health behaviour change in diabetes self-management, and health and social models of care. We noted significant differences for some behavioral outcomes for participants, but only one study reported an outcome in favour of the volunteer-based intervention for social support, although this may be because this outcome was not routinely measured in all studies. We highlight the need to focus more on the implementation of volunteer training, matching and retention, as well as intervention mode and duration, and geographical context. Further, other areas for future consideration include evaluation (comprehensively defining and measuring success) of volunteer programs and testing the effect of the intervention on volunteers who deliver the program, while exploring potential mechanisms of action within the causal pathway of improving social support and health. This work can be applied to future studies to develop volunteer programs and evaluate the implementation, impact, and effect of volunteers for social prescribing.

## Supporting information

S1 Checklist PRISMA checklist. (PDF)

S1 Table Search strategy and terms. The initial searches were completed in November 2023 and updated in May 2024.
(PDF)

S1 File All studies identified by the literature search. (XLSX)

S2 File All data extracted from the primary research sources. (XLSX)

S3 File Cochrane risk of bias (RoB) 2 results for each study. (XLSX)
