# Supplementary material for: A rapid systematic review of the effect of health or peer volunteers for diabetes self-management: Synthesizing evidence to guide social prescribing
Source: PLOS Glob Public Health. 2024 Dec 31;4(12):e0004071. doi: 10.1371/journal.pgph.0004071 (PMC11687712; doi:10.1371/journal.pgph.0004071)
Supplement: S1 Table — The initial searches were completed in November 2023 and updated in May 2024. (PDF) [file pgph.0004071.s002.pdf]

Database or Search Engine	Search Strategy
Medline, Embase, Cochrane Central Register of Controlled Trials<br><br>Ovid MEDLINE(R) and Epub Ahead of Print, In-Process, In-Data-Review & Other Non-Indexed Citations, Daily and Versions <1946 to May 15, 2024><br><br>EBM Reviews - Cochrane Central Register of Controlled Trials April 2024<br><br>Embase 1974 to 2024 May 15	1 *Community Health Workers/ or community health* work*.mp.<br>2 lay health worker.mp.<br>3 promotores.mp.<br>4 *Volunteers/ or volunteer*.mp.<br>5 peer.mp.<br>6 peers.mp.<br>7 1 or 2 or 3 or 4 or 5 or 6<br>8 Diabetes Mellitus, Type 2/ or diabetes.mp. or Diabetes Mellitus/<br>9 T2DM.mp.<br>10 8 or 9 770518<br>11 randomized controlled trial.m_titl.<br>12 7 and 10 and 11<br>13 limit 12 to yr="2013 -Current"
EBSCO Databases: APA PsycArticles, APA PsycInfo, CINAHL Complete, Social Work Abstracts, SPORTDiscus<br>May 16, 2024	(diabetes type 2 or diabetes mellitus type 2 or diabetes 2) AND (volunteers or community health workers or promotores or chws or lay health worker) AND (middle age older adults or elderly or geriatric or geriatrics or aging or senior or seniors or older people or aged 65 or 65+)
Epistemonikos<br>Primary study<br>May 16, 2024	(advanced_title_en:((advanced_title_en:(volunteer OR peer OR community health worker) OR advanced_abstract_en:(volunteer OR peer OR community health worker)) AND (advanced_title_en:(diabetes OR Type 2 OR T2DM) OR advanced_abstract_en:(diabetes OR Type 2 OR T2DM))) OR advanced_abstract_en:((advanced_title_en:(volunteer OR peer OR community health worker) OR advanced_abstract_en:(volunteer OR peer OR community health worker)) AND (advanced_title_en:(diabetes OR Type 2 OR T2DM) OR advanced_abstract_en:(diabetes OR Type 2 OR T2DM)))) AND (advanced_title_en:(randomized controlled trial) OR advanced_abstract_en:(randomized controlled trial))
Google Scholar<br>May 16, 2024	allintitle: diabetes peer OR lay OR volunteer OR "community health worker"-reviews -qualitative -"type 1" citations 2013-2024
Web of Science<br>May 16, 2024	1. volunteer OR peer OR community health worker (title) AND<br>2. diabetes OR T2DM Or Type 2 (title) AND<br>3. older adult OR middle-aged adult OR senior OR elderly OR aged (All fields)

The initial searches were completed in November 2023 and updated in May 2024
